# A Plea for Complete Differential Blood Count in All Original Examinations

**Published:** 1917-03

**Authors:** C. L. Maxwell

**Affiliations:** Myra, Texas


### A Plea for Complete Differential Blood Count in all Original Examinations.

BY DR. C. L. MAXWELL, MYRA, TEXAS.

*Mr. President and Gentlemen of the North Texas Medical Association:*

I come before you on this occasion not to discuss thoroughly the subject I have selected, but rather to suggest to you, if I can do so, the importance of using this branch of our knowledge in completing our original examination of all patients, more especially children. What I refer to is a complete differential blood count in all cases at first examination.

Long ages since, when plunged in the thickest night of ignorance and error, lay the world, save where in one small part called Greece where blazed the noonday sun of learning and art, which was destined to shed its beams into all time, Hippocrates became one of the great torch-bearers of civilization. This medical genius of that ancient clime formulated a theory as to the causation of disease, which is the embryonic framework of the differential blood count. He, in his empiric but philosophic reasoning, supposed that the human body was composed of four different humors—blood, phlegm, black bile and yellow bile. And that a disproportion in the normal percentages of one or more of these humors produced disease.

Working upon this hypothesis—that in disease there must be a disproportion in some of the elements of the body—Nasse, in 1835, sounded the keynote when he discovered and differentiated the white blood cells. Since the day of Nasse the classification of leucocytes has undergone many changes. The present most accepted method of classification divides them according to the shape of their nucleus, the presence or absence of granules in their protoplasm and the staining reaction of these granules.

Taking the form of nucleus as the basis of primal division, the leucocytes are divided into mono and polynuclear. The mononuclears are divided into granular and non-granular, and either of these divisions may take any one of the acid basic or neutral stains.

Accepting this form of nomenclature we have, in normal blood, of the mononuclear type:

| | |
|---|---|
| Small lymphocytes .............. | 15-23 per cent. |
| Large lymphocytes .............. | 7-11 per cent. |
| Transitional cells ............... | 2- 8 per cent. |

Then of the polynuclear variety:

| | |
|---|---|
| Neutrophiles ................... | 60-70 per cent. |
| Eosinophiles .................... | 2- 4 per cent. |
| Basophiles ..................... | |

Age produces quite a variation in these percentages. A child of 1 year has 30 per cent polynuclear neutrophiles and 56 per cent lymphocytes. At the age of 14 years they become as an adult. They vary between these ages directly as to the age of the child. In children the eosinophiles are ¼ per cent higher than in adults. So much for normal conditions.

In the interpretation of a blood count, we have to associate the differential count, as the total count alone is of little value. In the early days of hematology, undue prominence was accorded the total count, especially when it was high.

High leucocytosis does not always warrant operative intervention, nor does a leucopenia always signify a harmless condition. As a matter of fact, a typhoid or tubercular abscess may present no leucocytosis, and conversely a small boil may produce a high total white cell count. But a leucocytosis is significant because of the fact that it is the index of body resistance, not of the severity of the infection.

Important as is the leucocyte count, the differential count is of greater importance. Instead of a leucocytosis, a leucopenia may exist, yet a gangrenous appendicitis be discovered at operation. In such a condition there would be a marked rise in the percentage of the polynuclear neutrophiles. Therefore, the relative increase in polynuclear neutrophiles is not only an index to body resistance, but it also determines the degree of toxic absorption. For example, a total leucocytosis of 7500 in a case of appendicitis with a polynuclear percentage of 90, calls for immediate operative interference. While conversely a total count of 15,000 with a relative polynuclear neutrophilia of 80 per cent would mean that the infection was mild and the resistance good. Of course, in making such interpretations, the clinical history is of vast importance. The fever, however, is valueless as a guide in either diagnosis or prognosis in such conditions.

Despite its arbitrary basis, Gibson's chart gives excellent data in the border line cases. He observed that in cases of infection in which the resistance is good, the neutrophiles increased 1 per cent for every 1000 leucocytes over 10,000. He also assumed that 75 per cent of polynuclear neutrophiles and 10,000 leucocytes to be the upper limit of normal. Now, if the polynuclear neutrophiles rise faster than 1 per cent to each 1000 leucocytes, the infection is severe. If the polynuclear rise is less than 1 per cent to each 1000 leucocytes, then the infection is mild and the resistance is good. Thus we have an index to the patient's resistance and to the severity of the infection.

Passing from the general rules of surgical diagnosis, we will consider some of the most important individual cases, one of the most important being appendicitis, which, as an example, following the general rule, has been discussed. Practically all the acute abscesses present the blood picture just described.

Puerperal sepsis may show no leucocytosis. Leucopenia may be present if the leucoblastic tissues are paralyzed by the infection.

When a mild infection occurs in a patient of good resistance, there is usually a leucocytic reaction. The higher the leucocytosis the better the prognosis. In gonorrheal types the leucocytosis is low. In puerperal sepsis the red cells very often reveal the presence of a lethal agent in the blood when the leucocytes fail to give any suggestion of it. In this condition the leucoblastic tissues are paralyzed and red cells are used to fight the invading germs. This is a compensatory procession, the part of nature which is very expensive, as it uses the red cells so fast. The development of sudden anemia after parturition is evidence of puerperal sepsis. No other sepsis produces as marked an anemia so soon.

Syphilis presents a lymphocytosis as the chief hematological characteristic. There is also a marked secondary anemia. The severity of the infection may be gauged by the height of the lymphocytosis and the fall of the hemoglobin. In very severe syphilis the polynuclears may be reduced to 25 per cent. The eosinophiles may be increased in tertiary bone or skin lesions.

Drugs affect the blood picture to a certain extent. The iodides, quinine, salicylic acid, antipyrine, nuclein, digitalis and morphine all cause a leucocytosis. Ether has quite a hemolytic action. More than half the total reduction of hemoglobin occurs the first hour, reaching the lowest in twenty-four. Then, as a compensatory effort on the part of nature, the hematopoeitic tissues become overactive and a polycythemia results. DaCosta advises that no serious operation be done when the hemoglobin is below 50 per cent, though successful operations have been done when the hemoglobin was as low as 15 per cent. A leucocytosis always follows etherization. This is transient, however, seldom persisting over twenty-four hours.

Chloroform also produces a leucocytosis, but it does not cause such a hemolysis. It does reduce the coagulating power of the blood, thus exposing the patient to oozing or secondary hemorrhage.

In tuberculosis the blood picture is only changed as a result of complication, such as secondary infection or hemorrhage. The injection of tuberculin causes a leucocytosis, a lymphocytosis and an eosinophilia.

Abdominal colic can often be differentiated into its various forms by the blood count. Lead colic has as a characteristic a basic degeneration of the red cells. Nephrolithiasis and cholelithiasis produce no change in the blood unless there is an accompanying infection. In intestinal colic there is a leucocytosis, while in intestinal obstruction there is a high leucocytosis.

Carcinoma and ulcer of the stomach sometimes are hard to differentiate. In carcinoma there is a leucocytosis; in ulcer there is none. In carcinoma there are normoblasts (nucleated red blood cells); in ulcer these are rare. In carcinoma the digestive leucocytosis is absent; in ulcer it is present. In carcinoma the hemoglobin gradually decreases, while in ulcer it falls suddenly after hemorrhage but regenerates quickly.

In pernicious anemia and malignant disease the blood picture is of value in diagnosis. In malignant disease the color index is below 1. In pernicious anemia it is high (above 1). In malignant disease the normoblasts are more numerous than megaloblasts; in pernicious anemia the megaloblasts are more numerous. (Normoblasts are nucleated red cells of the ordinary size, and megaloblasts are the same but very large.) Malignant disease presents a leucocytosis; pernicious anemia none or a leucopenia. Malignant disease gives a polynuclear eosinophilia, and pernicious anemia a lymphocytosis.

Malaria is easy diagnosed if the plasmodium is discovered, but in case it is not found a mononuclear leucocytosis with a low red count marks the picture.

Grippe does not present a typical blood picture, but there is, however, a characteristic leukemia. In complications such as otitis, there is a leucocytosis. So when there is a leucocytosis in grippe it means a complication. The only difference in the hematology of grippe and typhoid fever is the absence of the Widal in the one and the presence of it in the other.

In diseases of the heart where compensation is taking place there is a high polycythemia. This is an effort on the part of nature to further the process of compensation. This increase in red cells is higher in the congenital than in the acquired form.

Pleurisy with effusion is accompanied by a leucocytosis of moderate degree, while if the effusion is purulent the leucocytosis is high.

My colleague, Dr. Roberts, and myself have made in the last thirty days over forty differential blood counts, and out of that number have found 14 eosinophilias ranging from 1.9 per cent to 9.5 per cent, and out of 10 fecal specimens examined we have found 8 that showed a parasitic infection of some form or other. We give you this personal work to illustrate to you the necessity of differential counts on all patients who live in our dear old Texas, where the boll weevil gets the cotton and the hookworm gets the babies. We also want to call your attention to the fact that in all our adult patients, where we were able to get a fecal specimen, we found parasitic infection. We also want to make the point that eosinophilia only suggests the need of a fecal examination.

Chronic skin diseases produce an eosinophilia. Eczema has 45 per cent, urticaria 30 per cent, lupus 7-12 per cent, pamphigus 33 per cent, psoriasis 20 per cent, trichiniasis 20 per cent, and hydatids of the liver 60 per cent. These are always accompanied by a relatively high leucocytosis.

I want to say, gentlemen, that I am not discussing eosinophilia *per se* but only as a guide to other conditions. I further want to call your attention to the fact that we do not know to what part eosinophilia plays in the makeup of our blood, either pathologically or physiologically, since we have the records of cases who state that they never felt better than when their eosinophile count was as high as 75 per cent. Now what good would a tonsillectomy, appendectomy or any other surgical operation do if we fail to recognize this parasitic infection? And might it not be one of the potent causes of a large per cent of post-operative cases who do not come back, so to speak, as we would like to see them do?

Gentlemen, we are all guilty more or less of the sin of omission, and some are guilty of the sin of commission. Can we not make this an occasion for some new resolutions, and give our patients all that is coming to them when they place themselves in our hands?

This paper does not present anything new on the subject, but is simply for the purpose of bringing out the simple things that can be done in hematology by the general practitioner. It is a plea for more scientific work and a more thorough investigation of our cases. It is our duty to our patients and to ourselves to make the science of medicine more practical and the practice of medicine more scientific. By so doing we enhance our power of diagnosis, prognosis and treatment, and are better enabled to study the profound and obscure pathology of disease. If the general practitioner would, with his unlimited opportunity for observation and clinical investigation, he could throw many a beam into the deep shadows of the dark caverns of clinical mystery.

---

Carry a fine handkerchief and then forget to cover your mouth when you cough.

